# Leukocyte Telomere Length Variability as a Potential Biomarker in Patients with PolyQ Diseases

**DOI:** 10.3390/antiox11081436

**Published:** 2022-07-24

**Authors:** Daniela Scarabino, Liana Veneziano, Alessia Fiore, Suran Nethisinghe, Elide Mantuano, Hector Garcia-Moreno, Gianmarco Bellucci, Nita Solanky, Maria Morello, Ginevra Zanni, Rosa Maria Corbo, Paola Giunti

**Affiliations:** 1 Institute of Molecular Biology and Pathology, National Research Council, 00185 Rome, Italy; 2 Institute of Translational Pharmacology, National Research Council, 00133 Rome, Italy; elide.mantuano@ift.cnr.it; 3 Department of Biology and Biotechnology, Sapienza University of Rome, 00185 Rome, Italy; alessia.fiore@uniroma1.it (A.F.); rosamaria.corbo@uniroma1.it (R.M.C.); 4 Ataxia Center, Department of Clinical and Movement Neuroscience, UCL Queen Square Institute of Neurology, University College, London WC1N 3BG, UK; s.nethisinghe@ucl.ac.uk (S.N.); h.garcia-moreno@ucl.ac.uk (H.G.-M.); nita.solanky@ucl.ac.uk (N.S.); p.giunti@ucl.ac.uk (P.G.); 5 Department of Neurosciences, Mental Health and Sensory Organs, Centre for Experimental Neurological Therapies (CENTERS), Sapienza University of Rome, 00185 Rome, Italy; gianmarco.bellucci@uniroma1.it; 6 Department of Experimental Medicine and Surgery, Tor Vergata University, 00133 Rome, Italy; morello@uniroma2.it; 7 Unit of Neuromuscolar and Neurodegenerative Disorders, Department of Neurosciences, Bambino Gesù Children’s Research Hospital, IRCCS, 00100 Rome, Italy; ginevra.zanni@opbg.net

**Keywords:** spinocerebellar ataxias, leukocyte telomere length, neurodegenerative diseases, biomarkers

## Abstract

SCA1, SCA2, and SCA3 are the most common forms of SCAs among the polyglutamine disorders, which include Huntington’s Disease (HD). We investigated the relationship between leukocyte telomere length (LTL) and the phenotype of SCA1, SCA2, and SCA3, comparing them with HD. The results showed that LTL was significantly reduced in SCA1 and SCA3 patients, while LTL was significantly longer in SCA2 patients. A significant negative relationship between LTL and age was observed in SCA1 but not in SCA2 subjects. LTL of SCA3 patients depend on both patient’s age and disease duration. The number of CAG repeats did not affect LTL in the three SCAs. Since LTL is considered an indirect marker of an inflammatory response and oxidative damage, our data suggest that in SCA1 inflammation is present already at an early stage of disease similar to in HD, while in SCA3 inflammation and impaired antioxidative processes are associated with disease progression. Interestingly, in SCA2, contrary to SCA1 and SCA3, the length of leukocyte telomeres does not reduce with age. We have observed that SCAs and HD show a differing behavior in LTL for each subtype, which could constitute relevant biomarkers if confirmed in larger cohorts and longitudinal studies.

## 1. Introduction

The spinocerebellar ataxias (SCAs) due to CAG expansion are the most common of the polyQ diseases along with Huntington’s Disease. The ataxias are a group of neurodegenerative disorders, which are clinically and genetically heterogeneous. So far, more than 40 genes and loci have been identified [1]. Seven SCAs are due to a CAG repeat expansion encoding for a polyglutamine stretch, while other SCAs are caused by non-coding repeat expansions, DNA deletions, or point mutations [2,3]. SCA1, SCA2, and SCA3 are the most common forms of SCAs, accounting for about one-half of all affected families [4]. The mean age at clinical onset for SCA1, SCA2, and SCA3 is the third or fourth decade of life [5,6]. The range of the normal and expanded CAG repeat is different for each form of SCAs. [3]. The genotype is a significant predictor of age at onset, which is inversely correlated with the number of the CAG repeats.

Clinically, SCA1, SCA2, and SCA3 share a set of features including cerebellar dysfunctions, which encompass gait ataxia, intention tremor, and eye-movement abnormalities [2]. Signs of sensorimotor or sensory neuropathy, consistent with peripheral nerve involvement, are usually present, together with pyramidal and extrapyramidal features [6].

From a neuropathological point of view, degeneration of Purkinje cells and cerebellar circuits are considered the predominant pathogenic mechanism for SCAs, underlying the characteristic ataxic symptoms [7]. However, heterogeneity in the neuropathology of SCAs is also emerging from different studies [1]. In fact, some SCAs result in a loss of more than 75% of the total Purkinje cell population, such as in SCA1 and SCA2, whilst in other SCAs the cell reduction is only 25%, as is the case of SCA3. There is often significant cerebellar and brain-stem degeneration, associated with a variable degree of damage to more rostral regions (such as the basal ganglia) and caudal regions (such as the spinal cord) [2].

The cause of the susceptibility of neurons in specific brain regions to the toxic effects of mutant proteins is still unclear, but it is becoming evident that the response of cells surrounding the neurons, such as glial cells, including microglia and astrocytes may play a role in SCA pathophysiology. This has been shown at least for SCA1 and SCA3. In SCA1, analysis of human autopsy material indicated a significant glial pathology [8], and, recently, it has been found that in mouse models, astrocytes and microglia are activated very early in SCA1 pathogenesis, and activation occurs specifically in the most severely affected regions of the brain [9,10]. In SCA3, an analysis of pons sections showed increased numbers of activated microglial cells as well as reactive astrocytes. Astrocytes and microglia activation, in turn, promotes an inflammatory response with overproduction of numerous reactive oxygen and nitrogen species and inflammatory cytokines, which could lead to neurodegeneration. In addition, for SCA3, an imbalance between reactive oxygen species (ROS) production and antioxidant defense has been observed, suggesting that greater oxidative stress in SCA3 cells plays an important role in the neurodegenerative process of the disease [11,12,13]. Oxidative stress, as well as mitochondrial dysfunction, has also been shown to be involved in the pathology of SCA2 [14].

Human telomeres consist of repeated TTAGGG nucleotide sequences present at the ends of chromosomes, where they play a protective role against DNA damage. During physiological DNA replication, telomeres progressively shorten with each cell division, due to the inability of the DNA polymerase to replicate the 3′-end of the DNA strand. Telomerase, a ribonucleoprotein complex, counteracts such shortening of the telomeres [15,16], but, although normally present during embryonic development, its activity is silenced in many human somatic tissues after birth [17]. Therefore, the telomeres progressively shorten in the replicating cells of adult tissues (including skin, kidney, liver, blood vessels, and peripheral leukocytes). This phenomenon is thought to indicate cellular age and reflect an organism’s biological age [18,19,20]. The onset of this replicative senescence in human cells has been reported to be triggered by the shortest telomeres [21,22]. However, in ~90% of human cancers, the normally silent human TERT gene (hTERT), encoding the human telomerase catalytic subunit, is activated or upregulated. In 10–15% of tumors, another DNA recombination mechanism, termed alternative lengthening of telomeres (ALT), reverses telomere attrition in order to bypass cellular senescence [23].

Due to tissue availability, the measurement of leukocyte telomere length (LTL) is also widely used in studies on aging and neurodegeneration, on the basis that telomere lengths from different tissues of the same individual are statistically correlated [24,25].

A number of epidemiological and population studies provided evidence that leukocyte telomere shortening is associated with aging [18,19,20,25] and with age-related chronic diseases, although some inconsistencies have been observed [26,27,28,29]. LTL was also investigated in the neurodegenerative diseases Alzheimer’s (AD), Parkinson’s (PD), and Huntington’s (HD) diseases and Friedreich’s ataxia (FRDA). Reduced LTL was frequently observed already in the prodromal stages of AD and HD [30,31], but no consistent evidence of shorter telomeres in PD was reported [32]. A common hallmark of AD, HD, and PD pathophysiology is the inflammatory response associated with microglia activation. This, in turn, will result in the subsequent production and release of cytokines promoting inflammation and ROS leading to oxidative stress [33]. The involvement of the peripheral immune system may promote leukocyte division and telomere shortening, and the overall rate of LTL reduction would depend on the importance of the inflammatory component in disease pathogenesis, together with the proportion of oxidative damage and its importance at different disease stages [30,31]. Moreover, telomere maintenance and mitochondrial function are intimately related and form a bidirectional, feedforward loop. Telomere damage leads to mitochondrial biosynthesis reprogramming and dysfunction, through activation of the tumor repressor gene p53 [34,35]. Conversely, mitochondrial dysfunction leads to telomere loss as well as telomere-dysfunction-induced foci (TIFs), in which DNA-damage-response factors are recruited to critically short and/or uncapped telomeres [36]. Interestingly, about 10–20% of the total cellular telomerase protein subunit hTERT is localized in mitochondria and shuttles between the nucleus and mitochondria [37]. A recent study showed that mitochondrial oxidative stress, in particular elevated superoxide levels and decreased hydrogen peroxide levels, induces telomere erosion in mutant mouse models of oxidative stress. This reduction in telomere length occurs despite an increase in telomerase activity and correlates with the onset of the disease phenotype [38]. Consequently, LTL measurement could be a useful biomarker for disease progression, reflecting the inflammatory component and oxidative stress in the various diseases or disease stages.

Among the CAG repeat disorders, LTL analysis has previously only been performed in HD, indicating that telomere shortening could be a useful biomarker for disease conversion in pre-manifest subjects [31]. Similar inflammatory processes and oxidative stress observed in HD have been reported in SCA1, SCA2, and SCA3. Therefore, the aim of our research was to analyze LTL as a marker of inflammation for SCA1, SCA2, and SCA3, in order to identify a potential biomarker for clinical onset and disease progression and compare our findings with our HD data. As in other neurodegenerative diseases, the study of LTL could provide information indirectly on the inflammatory/oxidative damage component in the pathophysiology of SCA and indicate the possible value of LTL as a biomarker of the various stages of the disease, which could be useful for new therapeutic-trials design.

## 2. Materials and Methods

### 2.1. Subjects

A cohort of 107 ataxic patients, molecularly diagnosed as SCA1 (n = 52), SCA2 (n = 26), and SCA3 (n = 29), collected at the Ataxia Centre, National Hospital for Neurology and Neurosurgery UCL/UCLH, London, UK (Ethical approval references: EUROSCA study, 04/Q0512/8; polyQ, 09/H0716/53), and at the Laboratory of Neurogenetics of the Institute of Translational Pharmacology of the CNR, Rome, Italy, was examined. In Table 1, the demographic and clinical characteristics of SCA1, SCA2, and SCA3 patients are reported. Clinical diagnosis was performed on the basis of the presence of gait ataxia, which generally is the first symptom identified in all the SCAs, with a small percentage of patients showing other symptoms at clinical onset [39,40].

The control subjects are blood donors recruited at the Department of Experimental Medicine and Surgery of the University of Tor Vergata, Rome. A sex- and age-matched control group was selected for each patient group, with two controls for each patient, with the exception of the SCA3 group, where two controls were selected for 86% of patients.

Written informed consent was obtained from all participants. DNA was collected for research purposes, according to the principles expressed in the Declaration of Helsinki.

### 2.2. Laboratory Methods

Genomic DNA was extracted from patient peripheral blood leukocytes using a FlexiGene DNA kit (QIAGEN, Hilden, Germany) in accordance with the instructions of the manufacturer.

Molecular diagnosis of the three SCA subtypes was performed according to Orr et al. [41] for SCA1, Pulst et al. [42] for SCA2, and Kawaguchi et al. [43] for SCA3.

Leukocyte telomere length was measured by monoplex real-time PCR quantitative analysis (monoplex qPCR) on a 7300 real-time PCR instrument (Applied Biosystems, Waltham, MA, USA). This method allows the determination of the number of copies of telomeric repeats (T) compared to a single-copy gene (S) used as a quantitative control (T/S ratio) [44]. The telomere and single-copy gene β-globin (HGB) were analyzed on the same plate in order to reduce inter-assay variability. DNA (35 ng) was amplified in a total volume of 20 μL containing 10 μL of SYBR Select Master Mix (Applied Biosystems); primers for telomeres and the single-copy gene were added to final concentrations of 0.1 μM (Tel Fw), 0.9 μM (Tel Rev), 0.3 μM (HGB Fw), and 0.7 μM (HGB Rev), respectively. The primer sequences were: Tel Fw 5′-CGGTTTGTTTGGGTTTGGGTTTGGGTTTGGGTTTGGGTT-3′; Tel Rev 5′-GGCTTGCCTTACCCTTACCCTTACCCTTACCCTTACCCT-3′; HGB Fw 5′-GCTTCTGACACAACTGTGTTCACTAGCAAC-3′; and HGB Rev 5′-CACCACCAACTTCATCCACGTTCACCTTGC-3′. The enzyme was activated at 95 °C for 10 min, followed by 40 cycles at 95 °C for 15 s and 60 °C for 1 min. In addition, two standard curves (one for HGB and one for telomere reactions) were prepared for each plate using a reference DNA sample (Control Genomic Human DNA, Applied Biosystems) diluted in series (dilution factor of 2) to produce 5 concentrations of DNA ranging from 50 to 6.25 ng in 20 μL. Measurements were performed in triplicates and are reported as a T/S ratio relative to the calibrator sample to allow comparison across runs. Replicate assays of the same sample were carried out to calculate the inter-assay variation. The average standard deviation over three different assays was 4.2%. Thus, assuming a normal distribution, samples differing in average telomere length by as little as 8.3% (1.96 × SD) should be distinguishable by this method at the 95% confidence interval [44]. No amplification of the negative controls with both primer sets (HGB and telomeres) was observed.

### 2.3. Statistical Analysis

Statistical analyses were performed using Statistix software (version 8.0; Analytical Software, Tallahassee, FL, USA). Parametric (ANOVA) and nonparametric (Kruskal–Wallis/Wilcoxon rank-sum test) tests were used to compare the distribution of quantitative variables between patients and controls and the distribution of the mean T/S ratio across age and repeat number classes. The level of significance was set at *p* < 0.05. The relationship between the T/S ratio and age at blood sampling was evaluated by regression analysis. When necessary, LTL was adjusted for age by multiple regression. A comparison of the regression lines slopes was carried out by a *t*-test. ROC curve analysis was performed through the easy ROC web interface. The optimal cut-point was identified according to the Youden Index method.

## 3. Results

### 3.1. Subjects

The demographic, genetic, and clinical features of the SCA1, SCA2, and SCA3 cohorts are summarized in Table 1.

The SCA1 group cohort consisted of 52 patients and 5 pre-symptomatic subjects. The linear-regression analysis between the number of CAG repeats in the expanded allele and age at onset was highly significant (*p* < 0.00001). The same highly significant correlation was found in 26 SCA2 patients, when CAG repeats and age at onset were analyzed using linear regression (*p* < 0.00001). For the 29 SCA3 subjects, the linear regression was highly significant (*p* = 0.002).

### 3.2. LTL Analysis

#### 3.2.1. SCA1

**Figure 1:**
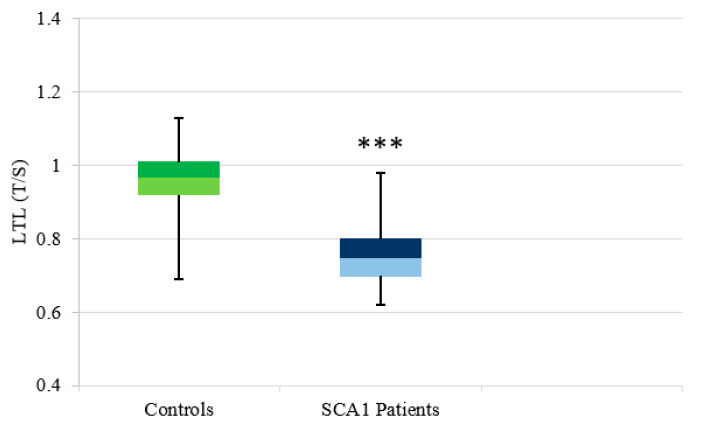
Distribution of LTL in controls and SCA1 patients. Box plot showing the distribution of LTL expressed as a relative telomere length T/S ratio (the number of copies of telomeric repeats T compared to a single copy gene S) used as a quantitative control. Comparison between control subjects, n = 104 (median: 0.97, Q1: 0.92, Q3: 1.01), and SCA 1 patients, n = 52 (median: 0.75, Q1: 0.70, Q3: 0.80), showed highly significant value (*** *p* < 0.001).

LTL, expressed as a T/S ratio, was measured in 52 SCA1 patients and 104 controls. We found that LTL was shorter in SCA1 patients (median: 0.75, Q1: 0.70, Q3: 0.80) compared to controls (median: 0.97, Q1: 0.92, Q3: 1.01; *p* < 0.00001) (Figure 1). In the five pre-manifest subjects, the median LTL value was 0.79 (Q1: 0.77, Q3: 0.95) and was significantly different both from controls (*p* = 0.03) and from SCA1 manifest patients (*p* = 0.04) (see Figure S1 in Supplementary Data).

No difference in mean LTL was observed between males and females in the two groups (controls: *p* = 0.20, SCA1 patients: *p* = 0.79).

**Figure 2:**
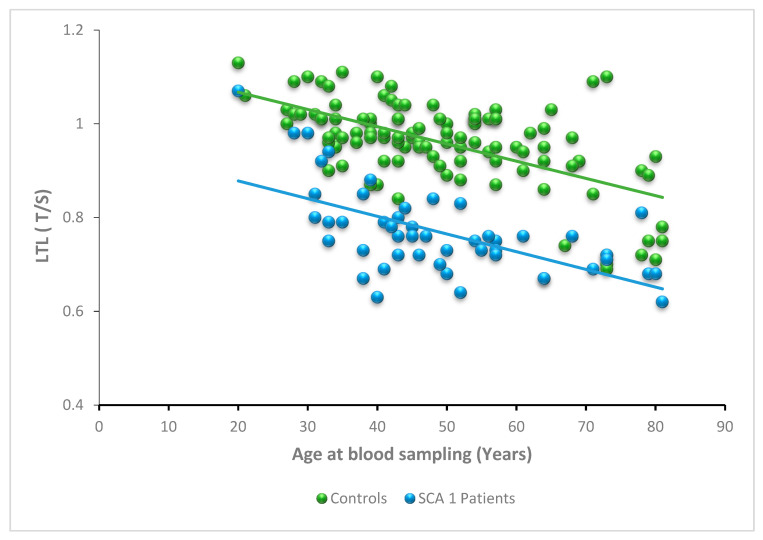
Relationship between LTL and age at blood sampling in SCA1 patients. LTL expressed as a relative telomere length T/S ratio as a function of age at blood sampling in SCA1 patients (n = 52; blue spheres) and controls (n = 104; green spheres). The LTL values of SCA1 patients are lower than controls at any age, as shown by the elevation of the regression lines (*p* < 0.0001). However, the slopes are similar (*p* = 0.88).

Linear-regression analysis showed an expected, significant negative relationship between LTL and age in the control group (y = −0.0037x + 1.14, *p* < 0.0001, R^2^ = 0.37). A significant relationship was observed between LTL and age in the SCA1 patient group as well (y = −0.0035x + 0.94, *p* < 0.0001, R^2^ = 0.34). The two regression lines show similar slopes (*p* = 0.88), but different elevations (*p* < 0.0001) indicate that from the age of about 20 years LTL of SCA1 patients and controls shows a similar shortening rate per year (0.0035 T/S vs. 0.0037 T/S), and SCA1 LTL values are lower than those of controls at any age (Figure 2), probably due to the early LTL shortening that began in premanifest SCA1 (Figure S1, Supplementary Data).

No relationship was observed between LTL values and CAG repeat number, after adjusting for age by means of multiple-regression analysis, (*p* = 0.15). The same result was observed between age-adjusted LTL and disease duration, which was calculated as the difference between age at blood sampling and the reported age of onset (*p* = 0.4).

We then assessed the accuracy of LTL as a biomarker of SCA1 disease through Receiver Operating Characteristic (ROC) curve analysis. In discriminating symptomatic SCA1 patients from controls, LTL displayed extremely high accuracy [AUROC(AUC) = 0.916, see Figure S2 in Supplementary Data]; setting the cut-off point of LTL at 0.85 allows for identifying SCA1 patients with 91.7% (80.0–97.7) sensitivity and 89.9% (82.7–94.9) specificity.

#### 3.2.2. SCA2

**Figure 3:**
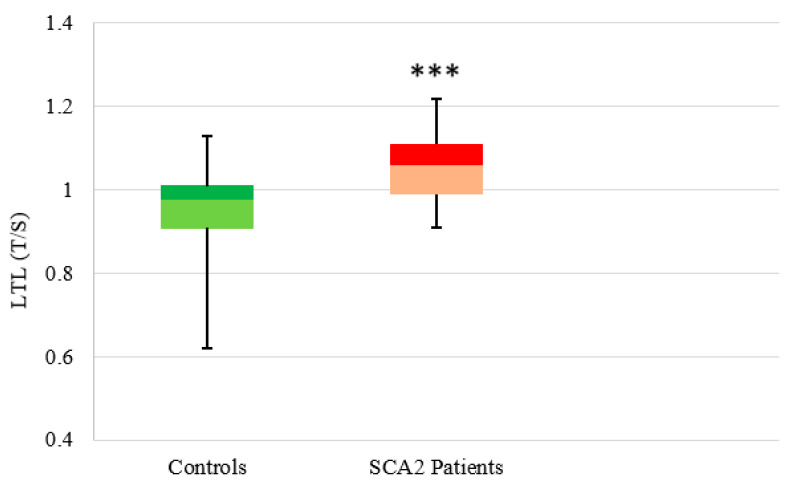
Distribution of LTL in controls and SCA2 patients. Box plot showing the distribution of LTL (T/S ratio) (the number of copies of telomeric repeats T compared to a single copy gene S) used as a quantitative measure in controls (n = 52) (median: 0.98, Q1: 0.91, Q3: 1.01) and SCA2 patients (n = 26) (median: 1.06, Q1: 1, Q3: 1.12) (*** *p* < 0.001).

A statistically significant difference was observed between the LTL distribution of 26 SCA2 patients (median: 1.06, Q1: 1, Q3: 1.12) and 52 controls (median: 0.98, Q1: 0.91, Q3: 1.01) (*p* < 0.00001), with longer telomeres in SCA2 patients (Figure 3).

No difference was observed in mean LTL between males and females in the two groups (controls: *p* = 0.28, SCA2 patients: *p* = 0.36).

**Figure 4:**
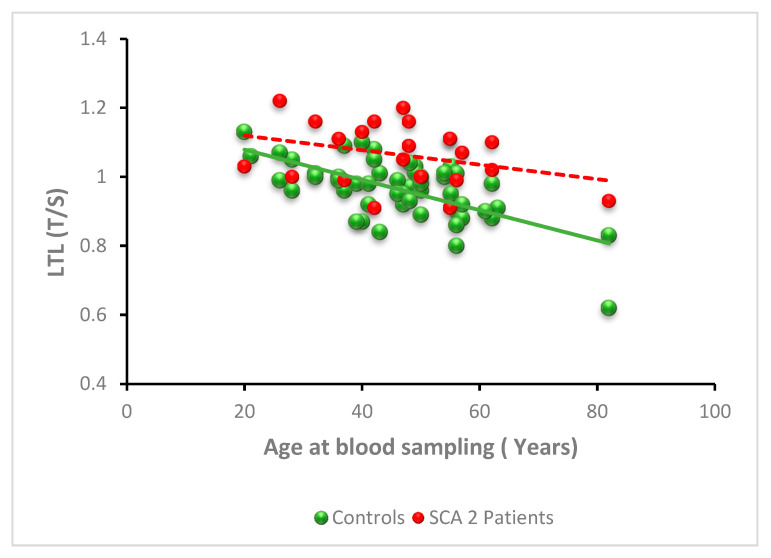
Relationship between LTL and age at blood sampling in SCA2 patients. LTL expressed as T/S ratio as a function of age at blood sampling in SCA1 patients (n = 26; red spheres) and controls (n = 52; green spheres). While in controls there is a significant negative correlation between LTL and age at blood sampling (*p* < 0.0001), in SCA2 this correlation is absent (*p* = 0.10). This is due to the maintenance of LTL in different ages. The LTL values of SCA2 patients are statistically significantly higher than controls from 40 years of age onwards.

Linear-regression analysis showed an expected significant negative relationship between LTL and age for the control group (y = −0.0040x + 1.17, *p* < 0.0001, R^2^ = 0.36), but, in SCA2 patients, the relationship between LTL and age was not statistically significant (y = −0.0020x + 1.16; *p* = 0.10) (Figure 4).

To investigate further, the relationship between age at blood sampling and LTL in SCA2 patients, we analyzed the LTL values for different age categories of SCA2 patients, compared to controls (Table 2). Again, LTL of control subjects showed a clear decreasing trend with age, while LTL of SCA2 patients was similar in all age classes. In addition, the median LTL of SCA2 patients was higher than controls in all age classes, and the difference became significant after 40 years, when LTL begins to shorten in control subjects but not in SCA2 patients (see Table 2).

No relationship was observed between LTL values and CAG number and disease duration, after adjusting for age by means of multiple-regression analysis (*p* = 0.23) and (*p* = 0.88), respectively. These results suggest that telomeres maintain a constant length throughout the duration of the disease.

We assessed the accuracy of LTL as a biomarker of SCA2 disease through Receiver Operating Characteristic (ROC) curve analysis. In discriminating symptomatic SCA2 patients from controls, setting the cut-off point of LTL at 0.99 [AUROC (AUC) = 0.792, see Figure S2B in Supplementary Data], allows for identifying SCA2 patients with 89.3% (71.8–97.7) sensitivity and 55.8% (41.3–69.5) specificity.

#### 3.2.3. SCA3

**Figure 5:**
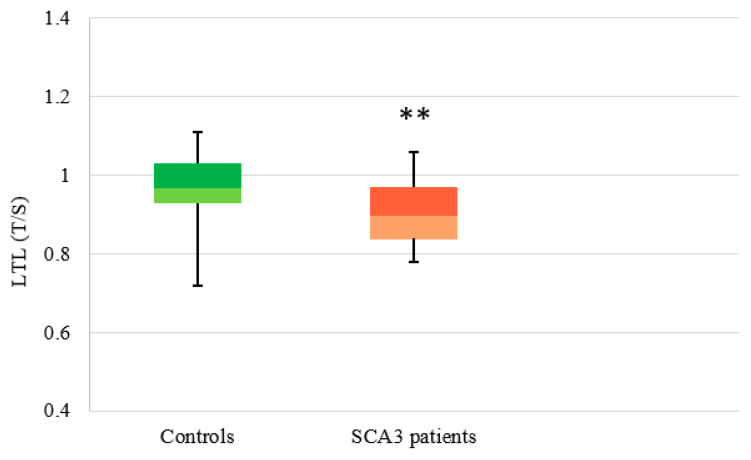
Distribution of LTL in controls and SCA3 patients. Box plot showing the distribution of LTL (T/S ratio) (the number of copies of telomeric repeats T compared to a single copy gene S) used as a quantitative measure in controls (n = 50) (median: 0.97, Q1: 0.93, Q3: 1.03) and SCA3 patients (n = 29) (median: 0.90, Q1: 0.84, Q3: 0.98) (** *p* < 0.01).

LTL was measured in 50 controls and in 29 SCA3 patients. A statistically significant difference was observed in the LTL distribution between controls (median: 0.97, Q1: 0.93, Q3: 1.03) and SCA3 patients (median: 0.90, Q1: 0.84, Q3: 0.98) (*p* = 0.003), with lower levels in SCA3 subjects (Figure 5).

There was no difference in LTL mean between males and females in the two groups (controls: *p* = 0.88, SCA3 patients: *p* = 0.11).

**Figure 6:**
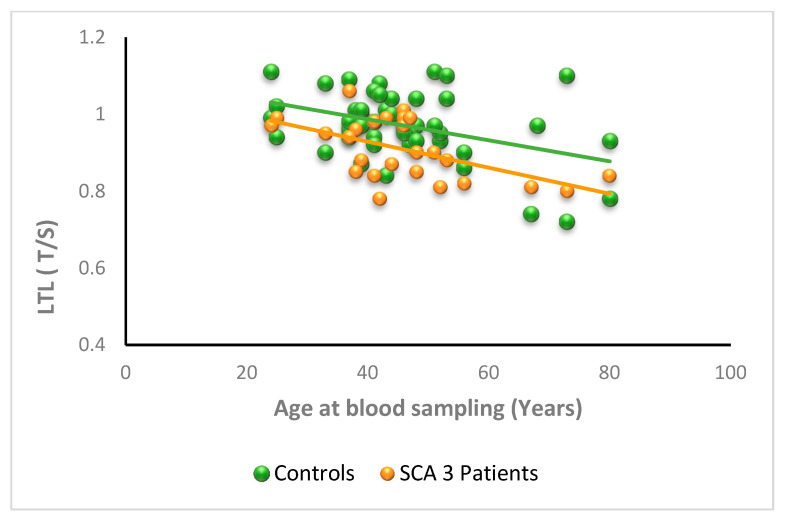
Relationship between LTL and age at blood sampling in SCA3 patients. LTL is expressed as the T/S ratio as a function of age at blood sampling in SCA3 patients (n = 29; orange spheres) and controls (n = 50; green spheres). The LTL values of SCA3 patients are lower than controls at any age (*p* = 0.003) The slopes of the lines are similar (*p* = 0.64), but the elevation differs (*p* = 0.007).

Linear-regression analysis showed the expected negative relationship between LTL and age in the control group (y = −0.0030x + 1.10, *p* =0.003, R^2^ = 0.17). A similar negative relationship of LTL with age at blood draw was observed for the SCA3 patient group (y = −0.0030x + 1.06, *p* = 0.003, R^2^ = 0.32). The two regression lines show similar slopes (*p* = 0.64) but different elevations (*p* = 0.007), indicating that from the age of about 20 years LTL of SCA3 patients and controls shows a similar shortening rate per year (both 0.003 T/S), and that SCA3 LTL values are lower than that of controls at any age (Figure 6). It is possible that in SCA3 too, an early LTL shortening begins in premanifest SCA3, although we have no real data to support this hypothesis.

**Figure 7:**
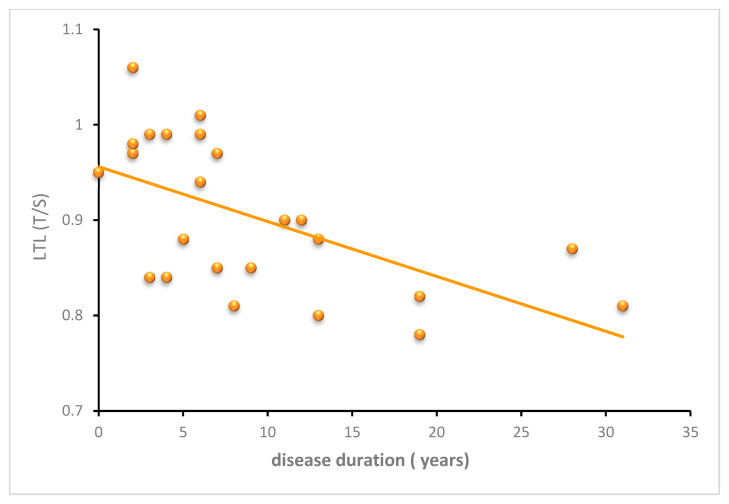
Relationship between LTL and SCA3 disease duration. Scatter plot showing the negative correlation between LTL and disease duration in SCA3 patients (y = −0.0057x + 0.96, *p* = 0.003).

No relationship was observed between LTL values and CAG number after adjusting for age by means of multiple-regression analysis (*p* = 0.60). The effect of disease duration on LTL was examined in SCA3 patients. A significant negative linear relationship between LTL and duration (y = −0.0057x + 0.96, *p* = 0.003) was observed (Figure 7), which was still significant even after adjusting for age by multiple-regression analysis (regression coefficient = −0.004; 95% CI: −0.008–0.0002, *p* = 0.02), suggesting that leukocyte telomeres shorten as the disease progresses, independent of age.

We assessed the accuracy of LTL as a biomarker of SCA3 disease through Receiver Operating Characteristic (ROC) curve analysis. In discriminating symptomatic SCA3 patients from controls, setting the cut-off point of LTL at 0.91 [AUROC(AUC) = 0.718, see Figure S2C in Supplementary Data], allows for identifying SCA1 patients with 57.7% (36.9–76.6) sensitivity and 84.0% (70.9–92.8) specificity.

## 4. Discussion

There is wide evidence that leukocyte telomere shortening is a common hallmark of conditions associated with increased systemic oxidative stress and chronic inflammation [45]. Previous studies on two neurodegenerative diseases, AD and HD, suggested that LTL could be a good biomarker of disease conversion from the prodromal stage to the full-blown disease [30,31,46,47,48]. Here, we extended the study to other polyQ diseases, SCA1, SCA2, and SCA3, which have not been explored to date.

Interestingly, despite these diseases being caused by mutations in three different genes, they all belong to the group of polyQ conditions [41,42,43], and LTL analysis shows a different behavior for each condition.

**SCA1**. Analysis of LTL in SCA1 patients showed significantly reduced values compared to controls. The analysis of a small number of pre-symptomatic SCA1 subjects suggests that the telomeric erosion could start at this stage. After clinical onset, telomere length depends on age but not on disease duration. The picture of LTL in SCA1 appears similar to what has been observed in HD [31,46], in which leukocyte telomeres begin to shorten in the pre-manifest stage (Supplementary Figure S1) and continue to do so in the manifest phase (Figure 1). However, when a comparison was made with HD, lower values of LTL were observed in HD patients [31] than in SCA1 patients. This distinguishes HD from SCA1.

The reduced LTL in SCA1 patients could suggest a state of peripheral inflammation secondary to neuroinflammation, as observed in other neurodegenerative diseases such as AD or HD [49]. Initial SCA1 studies focused on describing the neuronal pathology in SCA1, highlighting their dysfunction and degeneration. Further analysis of human autopsy material and studies on mouse models also showed a significant glial involvement in SCA1 pathology [8,9,10]. The authors described the activation of astrocytes and microglia, representing the brain’s immune cells, which are considered to be the primary mediators of neuroinflammation. LTL analysis in SCA1 appears to support the hypothesis that, besides neuronal pathology, the activation of microglia and neuroinflammation contribute to SCA1 pathogenesis. Furthermore, our observation that LTL shortening seems to begin in the pre-manifest stage of SCA1 is in line with the previous findings, showing glial activation occurring in the early stage of the disease in mouse models [9].

ROC curve analysis distinguishes SCA1 patients and controls in a sensitive and specific way (AUC 0.916), making LTL a possible biomarker useful for clinical trials to help stratification of SCA1 patients. In order to verify this as a potential biomarker for progression of the disease, a larger sample of patients should be recruited in a longitudinal study. The latter will be relevant for understanding the annual decrement in patients.

**SCA2**. The most relevant result of the LTL analysis in SCA2 is the observation of a significantly higher LTL in patients than in controls. This difference is more evident in those age groups in which LTL begins to decrease in controls, while LTL of SCA2 patients remains more stable. Furthermore, the LTL value seems independent from the CAG number and disease duration. The observation of longer telomeres in neurodegenerative disorders and in some forms of familial cancer is quite rare. Some familial cancers, caused by germline mutations in the TERT gene, are referred to as “long telomere syndromes”. Furthermore, somatic mutations of the TERT promoter are observed in 70% of solid tumors, indicating that telomerase abundance is critical for cancer initiation and progression [50,51]. Among neurodegenerative diseases, longer telomeres of leucocytes have been observed in Parkinson’s disease patients who develop dementia [52]. Amyotrophic lateral sclerosis (ALS) is another disease in which increased telomere length was reported, although this was not confirmed in all studies [53,54,55]. Since SCA2 alleles with intermediate expansions of 28–33 repeats may predispose to a higher risk of ALS [56], the result reported in this study of longer LTL in SCA2 patients appears to support the hypothesis of a common molecular substrate in the pathogenesis of PD with dementia, ALS, and SCA2. The presence of long telomeres compared to controls, although documented, is a phenomenon less clear than the telomere-shortening mechanism. The observation that telomerase expression was detectable in bone marrow mesenchymal stem cells (hMSC) derived from ALS patients but not in the healthy donor hMSC [53], may provide an explanation for the increased telomere length in ALS. However, this finding was contradicted by De Felice et al. [54], who reported a significantly lower telomerase-expression level in blood samples and spinal cord from ALS patients. To explain the longer telomeres in PD patients, it has been suggested that high levels of regulatory T cells (Tregs), which are known to suppress the immune response, could suppress the immune system with consequent reduction in cell divisions and, therefore, no shortening of the LTs [52]. Some studies also reported a neuroprotective effect of Tregs in PD [52]. In our view, these inconsistencies reflect the high heterogeneity in the etiology of these two syndromes, ALS and PD, conversely to SCA2. In SCA2, as there are no indications of neuroinflammation, it is possible that longer telomeres may reflect a reduced cell turnover due to a reduced immune response, or even one of the mechanisms observed for some ALS or PD, such as an enhanced telomerase expression.

**SCA3**. A different picture emerges from the analysis of LTL in SCA3. The LTL values of SCA3 patients were significantly reduced compared to controls, similarly to SCA1, but to a lesser extent. The telomere length of SCA3 patients was also found to depend on both patients’ age and disease duration. This suggests a possible different mechanism from SCA1, in which LTL is not dependent on either patient age or disease duration.

Involvement of the inflammatory system and dysregulation of cytokine expression has been reported in SCA3 [57,58,59,60], suggesting that this phenomenon may represent an early event in the pathophysiology of this condition [57]. In addition, it has been shown that ataxin-3 plays a protective role against cellular oxidative stress [11], and in vitro studies indicated an impaired antioxidative capacity in SCA3, which may promote increased susceptibility towards oxidative stress and neuronal cell death [61]. These findings were supported by in vivo studies, indicating that significant oxidative stress was only present after clinical onset [12]. The data as a whole suggest that the inflammatory phenomena inducing cell proliferation, together with the increase in oxidative stress, could explain the telomeric erosion observed in SCA3 and its increase as the disease progresses. Since the telomere length varies with the disease duration, the LTL value could be proposed as a clinical biomarker of disease progression. Longitudinal studies should be performed to corroborate this data.

The LTL trends and their relationship in SCA1, SCA2 and SCA3 are reported in Table 3.

## 5. Conclusions

We previously published the results of LTL in HD [31,46], and here we have compared that data with our new data for SCA1, SCA2, and SCA3. Interestingly, despite them all being PolyQ diseases, their LTL differs in each condition. We also correlated LTL with many different clinical variables.

**Figure 8:**
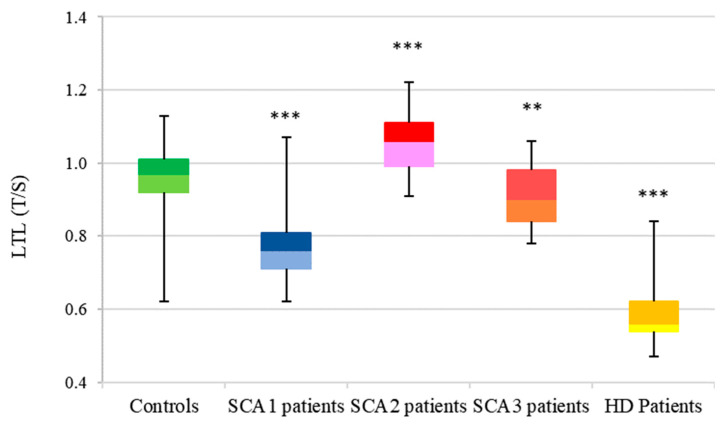
Distribution of LTL in CAG diseases. Box plot showing the distribution of LTL (T/S ratio) in controls (n = 146) (median: 0.97, Q1: 0.92, Q3: 1.01) and CAG repeat diseases: SCA1 patients (n = 52) (median: 0.75, Q1: 0.70, Q3: 0.80), SCA2 patients (n = 26) (median: 1.06, Q1: 1, Q3: 1.12), SCA3 patients (n = 29) (median: 0.90, Q1: 0.84, Q3: 0.0.98), and HD patients (n = 62) (median: 0.56, Q1: 0.53, Q3: 0.62). HD data from [31]. (** *p* < 0.01; *** *p* < 0.001 patients vs. controls). There are statistically significant differences between the four PolyQ diseases and controls.

In HD, SCA1, and SCA3, LTL was significantly shorter compared to controls. There was a variability of LTL among these three conditions, in which HD was significantly shorter than SCA1 and SCA3 (Figure 8, Table S1 in Supplementary Data).

In SCA1 and HD, we have a similar behavior in which the decrease in LTL could be a biomarker of the disease prodromal stage in SCA1 (Supplementary Figures S1 and S2), such as in HD [31], as they are shorter than controls. Moreover, in these two conditions, there was no correlation between LTL and disease duration. Future work on a greater number of pre-manifest SCA1 subjects will validate the possible use of LTL as a conversion biomarker. ROC analysis in SCA1 confirmed the sensitivity and specificity of LTL compared to controls, AUC 0.916 (0.864–0.969).

Conversely to HD and SCA1, SCA3 LTL was significantly negatively correlated with age and disease duration. A larger sample size and a longitudinal study could confirm SCA3 LTL as a promising biomarker of disease progression (Figure 7). Statistical analysis of ROC between SCA3 vs. controls showed a trend towards sensitivity and specificity, AUC 0.718 (0.596–0.839).

LTL is an indirect biomarker of inflammatory response and oxidative damage. This study provides support for the literature data elucidating the relevance of these mechanisms early in the disease process in SCA1 and HD [10,62], while in SCA3 inflammation and impaired antioxidative capacity are associated with disease progression [13]. In this context, the measurement of LTL appears to be a promising peripheral marker of the prodromal stages of SCA1 and of the progression of SCA3, especially in view of innovative therapies such as CRISPR/Cas9 and antisense oligonucleotides (ASOs), which have been recently developed in SCA preclinical models [63]. These developments highlighted the need for reliable biomarkers with adequate sensitivity to reflect disease progression or treatment responses. Measurement of LTL appears to be a peripheral marker, which fulfills these requirements, that could be added to clinical markers and improve the quality of the general assessment of disease evolution and/or therapy response.

In SCA2, the persistence of long telomeres in leukocytes is an intriguing observation that needs to be investigated to verify whether this is due to the persistence of telomerase, as observed in ALS patients [53], or to the suppression phenomena of the immune response, as proposed for Parkinson’s disease [52], or to the activation of the alternative lengthening of telomeres (ALT) mechanism, as shown in some tumors [23] and FRDA [64].

## Figures and Tables

**Table 1:** Demographic, genetic and clinical characteristics of SCA1, SCA2, and SCA3 patients and controls. For each patient, two age- and sex-matching controls were included in the study. Data are shown as mean ± standard deviation; NA: not applicable.

	SCA1		SCA2		SCA3	
	Patients N = 52	Controls N = 104	Patients N = 26	Controls N = 52	Patients N = 29	Controls N = 50
**Age at blood sampling (years)**	49.9 ± 15.2	49.1 ± 15.1	46.1 ± 12.3	46.4 ± 12.8	47.4 ± 14.3	46.04 ± 12.9
**Sex (males %)**	64.3	55.0	53.8	53.3	45.8	48.0
**CAG size in the expanded allele**	46.6 ± 5.7	NA	39.4 ± 2.2	NA	68.8 ± 4.3	NA
**Age at onset** **(years)**	43.8 ± 13.2	NA	37.0 ± 10.2	NA	39.7 ± 13.4	NA
**Disease Duration** **(years)**	7.6. ± 6.3	NA	9.2 ± 7.4	NA	9.6 ± 7.8	NA

**Table 2:** LTL (T/S) distribution by age at blood-sampling classes (median, Q1, and Q3 (n)). There is a significant difference between controls and SCA2 patients when age is ≥40 years.

Age Classes (Years)	Controls	SCA2 Patients	*p* Value
Total sample	0.98, 0.91–1.11 (52)	1.06, 0.99–1.11 (26)	<0.0001
19–29	1.06, 0.98–1.09 (6)	1.03, 1–1.22 (3)	0.90
30–39	1.00, 0.97–1.01 (8)	1.11, 1.02–1.15 (4)	0.20
40–49	0.98, 0.93–1.07 (18)	1.09, 1.02–1.16 (9)	0.009
>49	0.92, 0.88–1.0 (20)	1.01, 0.98–1.01 (10)	0.004
*p* value	0.009	0.39	

**Table 3:** Summary of the LTL trends and their relationships in SCA1, SCA2, SCA3, and HD. Summary of the LTL trends (patients vs. controls) in SCA1, SCA2, SCA3, and HD [31], and their relationships with age, CAG repeat number, and disease duration. The symbol of the arrows indicates the decrease and the increase in LTL, +/− indicate the presence/absence of the relationship.

	LTL Patient vs. Controls	LTL/Age Relationship	LTL/ CAG Repeat n° Relationship	LTL/Disease Duration Relationship
**SCA1**	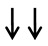	+	−	−
**SCA2**	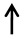	−	−	−
**SCA3**	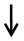	+	−	+
**HD**	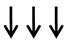	+	−	−

## Data Availability

The data presented in this study are available on request from the corresponding authors. Data are not publicly available due to privacy restrictions.

## Supplementary Materials

The following are available online at https://www.mdpi.com/article/10.3390/antiox11081436/s1. Figure S1: Distribution of LTL in controls, SCA1 pre-manifest and manifest subjects. Table S1: Statistical analysis of each cohort of patients (SCA1, 2, 3 and HD vs controls and vs. each other. Figure S2: Assessment of LTL as a biomarker of SCAs.
