# Supplementary material for: Leukocyte Telomere Length Variability as a Potential Biomarker in Patients with PolyQ Diseases
Source: Antioxidants (Basel). 2022 Jul 24;11(8):1436. doi: 10.3390/antiox11081436 (PMC9332235; doi:10.3390/antiox11081436)
Supplement: Supplementary file 1 [file antioxidants-11-01436-s001.zip › antioxidants-1798081-supplementary.pdf]

Supplementary Figures:

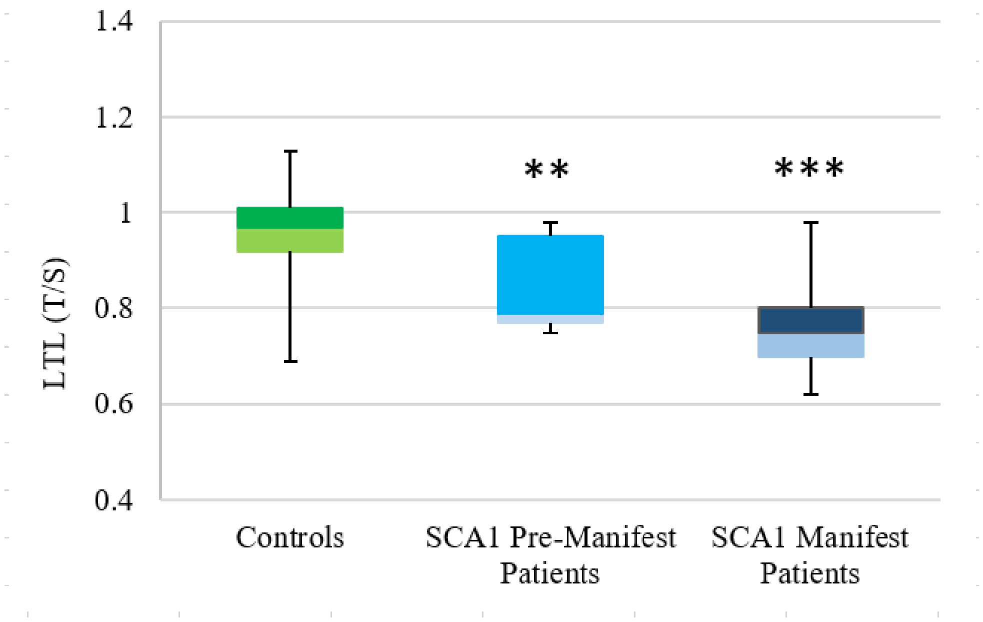

Supplementary Figure S1. **Distribution of LTL in controls, SCA1 pre-manifest and manifest subjects.** Box Plot showing the distribution of LTL (T/S ratio) in controls (median:0.97, Q1:0.92, Q3:1.01), SCA1 pre-manifest patients (median 0.79, Q1:0.77, Q3:0.95) and manifest patients (median:0.75, Q1:0.70, Q3:0.80). (**p<0.01; ***p<0.001)

	**SCA1**	**SCA2**	**SCA3**	**HD**
**CONTROLS**	p<0.001	p<0.001	p= 0.003	p<0.0001
**SCA1**		p<0.001	p=0.002	p<0.001
**SCA2**			p<0.001	p<0.0001
**SCA3**				p<0.001

Supplementary Table S1 Statistical analysis of each cohort of patients (SCA1,2, 3 and HD vs controls and vs each other. The analysis was carried out using Kruskal-Wallis method, adjusted by Bonferroni error correction.

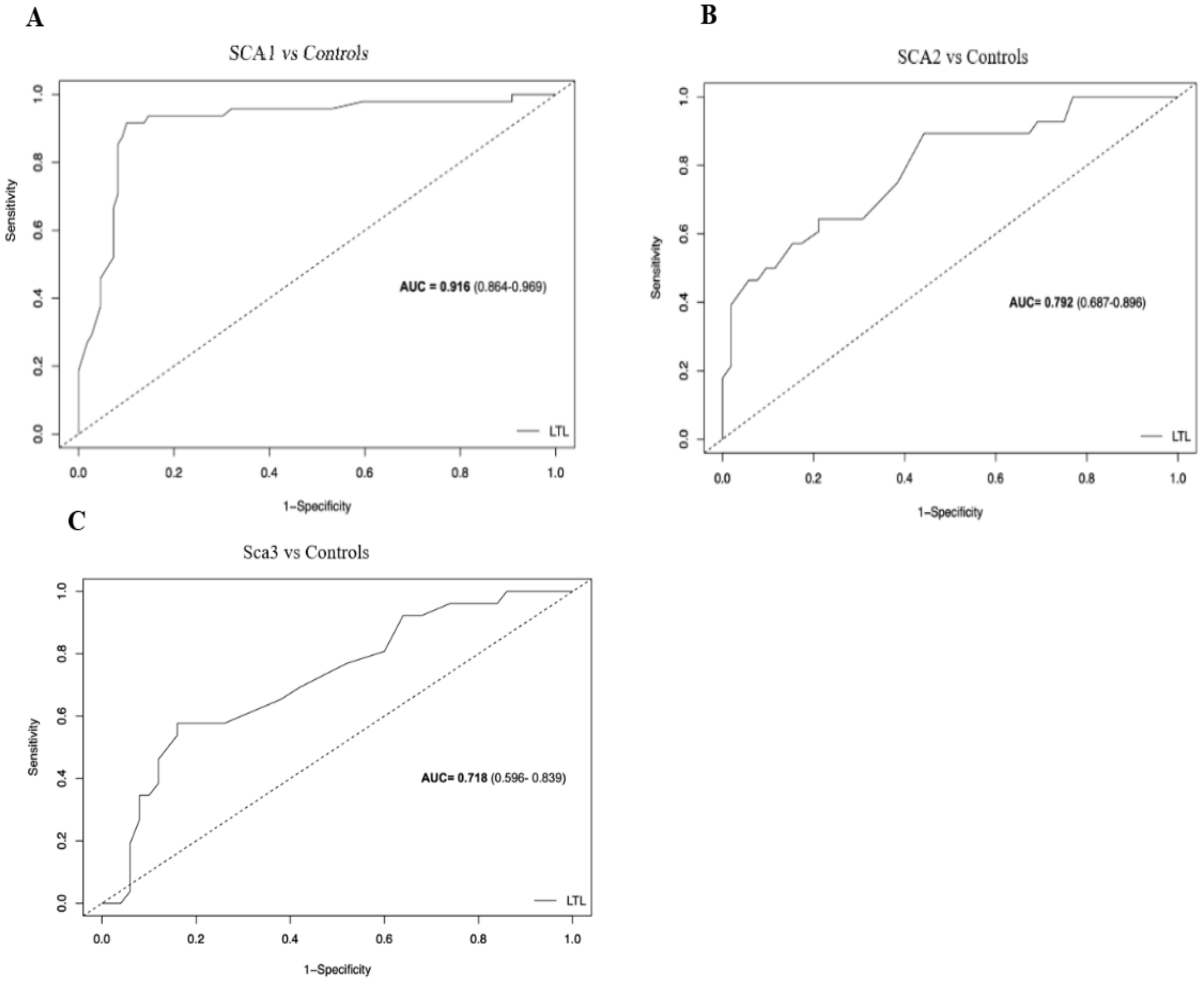

Supplementary Figure S2: **Assessment of LTL as a biomarker of SCAs.** ROC curves for discrimination between patients and controls: (A) SCA1, (B) SCA2, (C) SCA3
